# Dietary AhR Ligands Regulate AhRR Expression in Intestinal Immune Cells and Intestinal Microbiota Composition

**DOI:** 10.3390/ijms21093189

**Published:** 2020-04-30

**Authors:** Oliver Schanz, Rieka Chijiiwa, Sevgi Can Cengiz, Yasmin Majlesain, Heike Weighardt, Haruko Takeyama, Irmgard Förster

**Affiliations:** 1 Immunology and Environment, Life and Medical Sciences (LIMES) Institute, University of Bonn, Carl-Troll-Straße 31, 53115 Bonn, Germany; oliver.schanz@uni-bonn.de (O.S.); secen102@hhu.de (S.C.C.); yasmin_majlesain@uni-bonn.de (Y.M.); 2 Department of Life Science and Medical Bioscience, Graduate School of Advanced Science and Engineering, Waseda University, 2-2 Wakamatsucho, Shinjuku-ku, Tokyo 162-8480, Japan; rieca.chijiiwa@asagi.waseda.jp; 3 Computational Bio Big-Data Open Innovation Laboratory (CBBD-OIL), National Institute of Advanced Industrial Science and Technology, 3-4-1 Okubo, Shinjuku-ku, Tokyo 169-8555, Japan; 4 Research Organization for Nano and Life Innovation, Waseda University, 513 Wasedatsurumaki-cho, Shinjuku-ku, Tokyo 162-0041, Japan; 5 Institute for Advanced Research of Biosystem Dynamics, Waseda Research Institute for Science and Engineering, Graduate School of Advanced Science and Engineering, Waseda University, 3-4-1 Okubo, Shinjuku-ku, Tokyo 169-8555, Japan

**Keywords:** mucosal immunity, colitis, microbiome, aryl hydrocarbon receptor, aryl hydrocarbon receptor repressor, phytochemicals

## Abstract

A diet rich in vegetables and fruit is generally considered healthy because of a high content of phytochemicals, vitamins, and fiber. The phytochemical indole-3-carbinol (I3C), a derivative of glucobrassicin, is sold as a dietary supplement promising diverse health benefits. I3C metabolites act as ligands of the aryl hydrocarbon receptor (AhR), an important sensor for environmental polyaromatic chemicals. Here, we investigated how dietary AhR ligand supplementation influences AhR target gene expression and intestinal microbiota composition. For this, we used AhR repressor (AhRR)-reporter mice as a tool to study AhR activation in the intestine following dietary I3C-supplementation in comparison with AhR ligand-deprived diets, including a high fat diet. AhRR expression in intestinal immune cells was mainly driven by dietary AhR ligands and was independent of microbial metabolites. A lack of dietary AhR ligands caused enhanced susceptibility to dextran sodium sulfate (DSS)-induced colitis and correlated with the expansion of *Enterobacteriaceae*, whereas *Clostridiales*, *Muribaculaceae*, and *Rikenellaceae* were strongly reduced. I3C supplementation largely reverted this effect. Comparison of I3C-induced changes in microbiota composition using wild-type (WT), AhRR-deficient, and AhR-deficient mice revealed both AhR-dependent and -independent alterations in the microbiome. Overall, our study demonstrates that dietary AhR ligand supplementation has a profound influence on *Ahrr* expression in intestinal immune cells as well as microbiota composition.

## 1. Introduction

Environmental signals, such as dietary, microbial, or xenobiotic factors, are sensed in the intestinal tissue by the aryl hydrocarbon receptor (AhR), which is an important regulator of metabolism, but also influences immune cell homeostasis and immune activation in the intestine [1]. AhR expression is especially high in the liver and in barrier tissues such as skin, lung, and gut [2]. Additionally, it has been shown that the AhR can be induced by immunological stimuli, such as pathogen-associated molecular patterns [1].

AhR activation plays an important role in intestinal immunity, contributing to intestinal homeostasis, inflammation, and host defense [3,4]. The AhR controls interleukin (IL)-22 production by innate lymphoid cells (ILCs), and thus confers host defense against *Citrobacter rodentium* infection in mice [3]. Besides IL-22 production, AhR activation through high affinity AhR ligands has been shown to stimulate the production of antimicrobial peptides and regulate tissue regeneration [5]. AhR-deficient mice are highly susceptible to dextran sodium sulfate (DSS)-induced colitis, which at least in part results from a reduction of intraepithelial lymphocytes (IELs) [6,7] and impaired homeostasis of intestinal epithelial cells (IEL) [8]. Furthermore, the AhR has been shown to be an important regulator of T cell immunity in intestinal inflammation, regulating IL-17, Foxp3, IL-10, and IL-22 expression, and altogether ameliorating colitis symptoms and maintaining intestinal homeostasis [9,10,11]. In addition, Monteleone et al. could show that the AhR is down-regulated in intestinal tissue of patients with inflammatory bowel disease (IBD) and that AhR signalling is able to inhibit inflammation in colitis of the gastrointestinal tract of mice [12]. This indicates a major role of the AhR in resolving intestinal inflammation and makes the AhR an interesting pharmacological target in IBD.

One of the key target genes activated by the genomic AhR pathway is the AhR repressor (AhRR). The AhRR is highly homologous to the AhR, but lacks the Period/ARNT/Single-minded (PAS)-B and the transactivation domain. It competes with AhR for AhR nuclear translocator (ARNT) binding, but cannot initiate transcription, and hence suppresses the AhR signalling pathway [13,14,15,16]. Additionally, it has been demonstrated that the AhRR may interfere with non-canonical AhR-mediated signalling by interacting with RelB, and thereby inhibiting inflammatory responses [17]. *Ahrr* expression is restricted to some cell populations in a given tissue and does not strictly correlate with expression of *Cyp1a1*, *Cyp1a2*, and *Cyp1b1*, other major target genes of the AhR encoding important cytochrome p450 enzymes, which are responsible for degradation of AhR ligands [2,14,18,19,20]. Specifically, using AhRR/Enhanced Green Fluorescent Protein (EGFP) reporter mice, we could show that *Ahrr* expression is restricted to immune cells in barrier organs, such as skin and intestine [21]. We could further observe that AhRR activation is indeed largely AhR-dependent and that cell types that highly express the *Ahrr* display only mild *Cyp1a1* expression and vice versa, indicating the importance of different feedback inhibition mechanisms in different cell types. Interestingly both, AhR and AhRR-deficient mice are highly susceptible to DSS-induced colitis, which is likely due to the highly cell type specific expression of the AhRR and the resulting cell type-specific differences in AhR/AhRR signalling [21].

The mucosal surface area of the gut represents an enormous area, which is in direct contact with the environment. In addition to occasional pathogen encounters, the intestinal immune system is constantly exposed to antigens from the diet or the microbiota. Therefore, it is essential that gut-associated immune cells maintain a balance between protection against harmful infections and tolerating harmless food-derived antigens and commensals. Constant availability of dietary substances and the microbiota in the intestinal lumen lead to continuous stimulation of the AhR signalling pathway. With regard to food, cruciferous vegetables are an important source of AhR ligands in the intestine as they contain high concentrations of glucobrassicin. Enzymatic processing of this substance leads to the formation of indole-3-carbinol (I3C). Stomach acid-catalysed condensation of I3C then generates a number of biologically active substances, such as the AhR ligands 3,3′-diindolylmethane (DIM) and indolo[3,2-b]carbazole (ICZ) [6]. Dietary supplementation of rodents with I3C [6,22] or broccoli extracts containing glucobrassicin [23] led to profound changes in the microbiome and conferred protection from DSS-induced colitis. Next to dietary components, microbial factors are able to activate AhR-signalling. The AhR senses bacterial pigments, leading to induction of canonical detoxifying genes as well as regulation of cytokines and chemokines, thereby fighting bacterial infections [24]. Furthermore, tryptophan derivatives derived from the diet or bacterial metabolites have been shown to tune the intestinal immune system by signalling through the AhR [5,25]. The availability of microbiota produced AhR ligands is critically dependent on the local microbiota composition and the presence of certain bacterial strains. *Lactobacillus reuteri*, which has the ability to metabolize tryptophan to AhR activating indoles, and the probiotic strain *Lactobacillus bulgaricus* OLL1181, have shown AhR activating potential [26,27].

In this study, we analyzed the influence of dietary AhR ligands on AhRR expression, colitis pathology, and changes in the microbiome. Using AhRR-reporter mice, we could show that AhR activation in intestinal immune cells is modulated by dietary AhR ligands, but not by the absence of commensal microbes. The application of several AhR-ligands in normal mouse chow or supplementation of a ligand-reduced diet (LRD) with I3C protected from DSS-induced colitis and significantly changed the microbial community in the gut compared with LRD. We provide insight into the interplay of gut microbiota and AhR signalling and demonstrate that dietary intervention with I3C acts in an AhR-dependent as well as AhR-independent manner.

## 2. Results

### 2.1. Dietary AhR Ligands Strengthen Intestinal Barrier Integrity and Lower Susceptibility to Colitis

**Figure 1:**
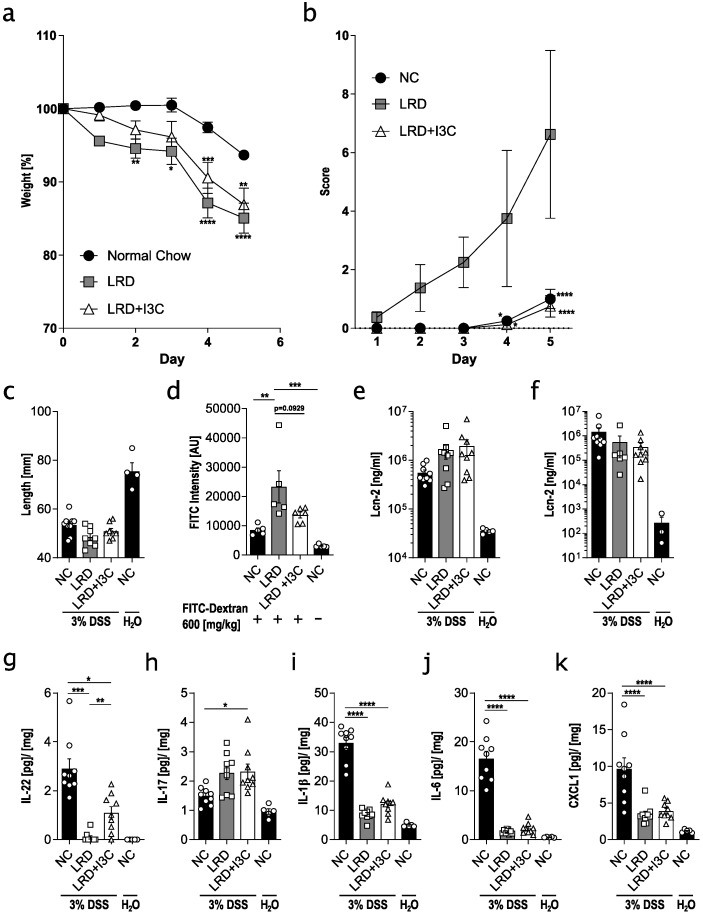
Dietary aryl hydrocarbon receptor (AhR) ligands influence the intestinal barrier integrity and susceptibility to dextran sodium sulfate (DSS)-induced colitis. C57BL/6 mice were fed for four weeks from weaning onwards with normal chow (NC), AhR ligand reduced diet (LRD), or LRD supplemented with indole-3-carbinol (LRD + I3C) (2 g/kg). Afterwards, mice were treated with 3% DSS via the drinking water for five days. Over the course of the experiment, body weight was monitored (**a**) and disease activity score (**b**) composed of general condition, spontaneous behavior, posture, and body weight was determined. (**c**) Colon length was measured at the end of the experiment. (**d**) Mice were fasted for four hours and orally gavaged with 600 mg/kg fluorescein isothiocyanate (FITC)-dextran, and FITC fluorescence in the serum was determined four hours later. Lipocalin-2 (LCN2) levels were measured in serum (**e**) and stool lysates (**f**) of DSS-treated mice and untreated control mice under different dietary conditions. Protein lysates of colonic tissue were analyzed for levels of interleukin (IL)-22 (**g**), IL-17 (**h**), IL-1β (**i**), IL-6 (**j**), and CXCL1 (**k**). Data are pooled from at least two independent experiments (*n* = 3–17). Results are shown as mean +/− SEM and significance was analyzed by ordinary one-way analysis of variance (ANOVA) or two-way ANOVA (a/b) (* *p* < 0.05, ** *p* < 0.01, *** *p* < 0.001, **** *p* < 0.0001).

To analyze the influence of dietary AhR ligands on colitis susceptibility and intestinal barrier integrity, we fed C57BL/6 mice for four weeks from weaning onwards with different diets and treated mice subsequently for five days with 3% DSS in the drinking water. Mice that received normal chow (NC) showed the least weight loss (Figure 1a) and the lowest disease score (Figure 1b), but their colon was clearly shortened compared with control mice that were not treated with DSS (Figure 1c), indicative of induction of colitis. Mice fed with the AhR ligand reduced diet AIN-93G (LRD) lost significantly more weight, had a higher disease score, and displayed slightly enhanced colon shortening compared with NC-fed mice. When LRD was supplemented with 2 g/kg I3C (LRD + I3C), the disease score was similar to that of NC fed mice, while weight loss was less severe compared with LRD-fed mice, in line with previously published results [6]. Colon shortening, however, was not significantly different compared with NC- and LRD-fed mice (Figure 1c). As AhR signalling influences the intestinal barrier integrity [6,22], we assessed barrier function by oral application of fluorescein isothiocyanate (FITC)-Dextran after feeding mice for four weeks with either NC, LRD, or LRD + I3C. Mice fed with NC showed the lowest degree of FITC-Dextran transmission into the circulation (Figure 1d), while mice fed with LRD had a significantly higher FITC-Dextran transmission, indicating a disturbed barrier integrity. LRD + I3C reduced FITC-Dextran transmission, demonstrating that AhR activation, through either a variety of phytochemicals in NC, or a defined group of ligands in LRD + I3C, strengthens the intestinal barrier. To further assess the severity of DSS colitis in mice fed with different diets, fecal and serum levels of lipocalin-2 (LCN2), a biomarker for intestinal inflammation [28], were determined. Both fecal and serum LCN2 levels were elevated in mice treated with DSS compared with untreated control mice (Figure 1e,f), but no differences between NC, LRD, and LRD + I3C fed mice could be detected. It has been shown that disease severity in colitis correlates with higher levels of proinflammatory cytokines [29]. Cytokine levels were determined in protein lysates of colonic tissue after five days of DSS treatment. As expected, expression levels of IL-22, IL-1β, IL-6, and CXCL1 were clearly enhanced in the colonic tissue of mice fed NC after DSS treatment compared with untreated mice (Figure 1g–k). IL-22 was not induced in mice fed LRD, but moderate levels of IL-22 were observed in mice fed LRD + I3C. IL-1β, IL-6, and CXCL1 were significantly reduced in DSS-treated mice after feeding LRD and LRD + I3C as compared with NC-fed mice. In contrast, an elevation of IL-17 was observed in LRD and LRD + I3C fed mice after DSS treatment, whereas no difference in IL-17 levels was detected for control and DSS-treated mice fed NC. Taken together, these data confirm that dietary AhR ligands ameliorate susceptibility to colitis and show that IL-22, which is critical for barrier homeostasis and also represents an AhR target [30], inversely correlates with colitis severity.

### 2.2. Dietary AhR Ligands Drive Expression of the AhRR in Intestinal Immune Cells

**Figure 2:**
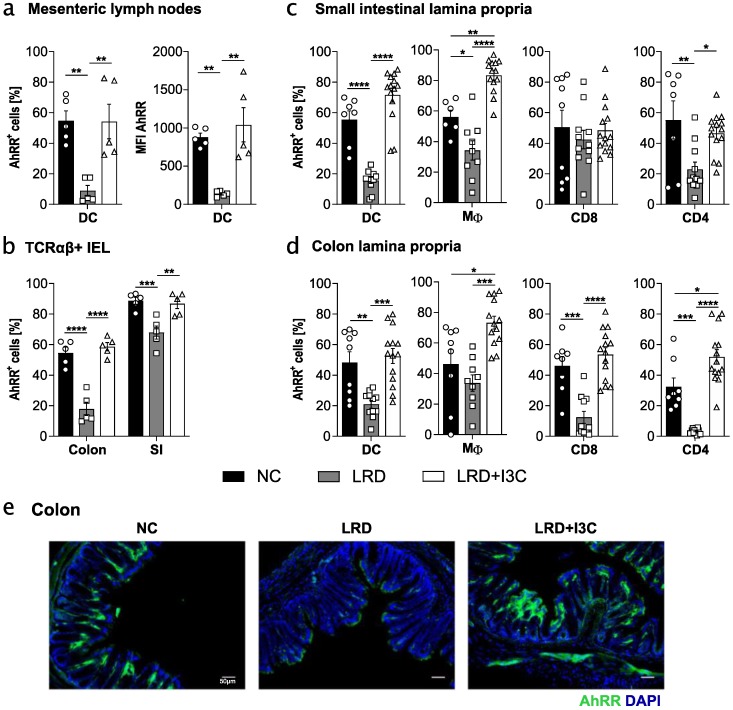
Dietary AhR ligands drive expression of *Ahrr* in intestinal immune cells. AhRR^E/+^ mice were Figure 3. carbinol (LRD + I3C) (2 g/kg). Frequency of *Ahrr* expressing dendritic cells (DCs) and mean fluorescent intensity (MFI) of DCs in the mesenteric lymph nodes (mLNs) (**a**); frequency of *Ahrr* expressing T cell receptor (TCR)αβ^+^ IELs (intraepithelial lymphocytes) in colon and small intestine (SI) (**b**); and frequency of *Ahrr* expressing DCs, MΦ, CD4^+^ T cells, and CD8^+^ T cells isolated from the lamina propria of the SI (**c**) and colon (**d**). (**e**) After paraformaldehyde (PFA) fixation, cryosections of the colon were stained with DAPI in addition to the endogenous Enhanced Green Fluorescent Protein (EGFP) reporter. Data are pooled from at least two independent experiments (*n* = 5–14), scale bar: 50 µm. Results are shown as mean +/− SEM and significance was analyzed by one-way ANOVA corrected for multiple comparisons by the Tukey’s post hoc test (* *p* < 0.05, ** *p* < 0.01, *** *p* < 0.001, **** *p* < 0.0001). Pictures are representative of at least three different mice from at least two independent experiments. DAPI = 4′,6-Diamidin-2-phenylindol.

We have previously shown that the expression of the AhRR is mostly dependent on environmentally induced AhR signalling [21]. Therefore, we used AhRR/EGFP reporter expression in intestinal immune cells as readout for AhR activation through dietary AhR ligands. We fed AhRR^E/+^ mice for four weeks with NC, LRD, or LRD + I3C, and determined the frequency of AhRR/EGFP^+^ intestinal immune cells. A high frequency of AhRR/EGFP^+^ dendritic cells (DCs) in mesenteric lymph nodes (mLNs) could be detected when mice were fed with NC or LRD + I3C, whereas the frequency of these cells was significantly reduced in LRD-fed mice (Figure 2a).

This could also be observed when measuring the mean fluorescent intensity (MFI) of the EGFP reporter in mLN DCs, indicating that not only frequencies of *Ahrr*-expressing cells, but also the intensity of the AhRR/EGFP expression, was regulated by dietary AhR ligands. T cells and B cells in the mLNs showed no *Ahrr* expression, as previously published (not shown) [21]. Moreover, the proportion of AhRR^+^ T cell receptor (TCR)αβ^+^ colonic IELs was significantly reduced in mice fed LRD and was enhanced after feeding LRD + I3C. This effect was less pronounced in the small intestine (SI) (Figure 2b). The MFI of the AhRR/EGFP signal was generally higher in the SI and a reduction of the MFI in LRD-fed mice was strongly visible in both the colon and SI (Figure S1a). These observations were largely the same for TCRγδ^+^ IELs (Figure S1b). In the lamina propria of the SI and colon, a similar reduction of *Ahrr* expression in LRD-fed mice and a rise after feeding LRD + I3C could be observed for most cell populations (Figure 2c,d). CD8^+^ T cells in the SI lamina propria of LRD fed mice, however, showed no reduction in *Ahrr* expression and intensity (Figure 2c and Figure S1c). In some cases, namely macrophages in the lamina propria of SI and colon and CD4^+^ T cells in the colonic lamina propria, feeding LRD + I3C led to even higher frequencies of AhRR/EGFP^+^ cells compared with NC-fed mice (Figure 2c,d). The MFI of the AhRR/EGFP reporter was notably higher in all lamina propria immune cell subsets of LRD + I3C fed mice compared with NC-fed mice (Figure S1c,d). Histological analysis of AhRR/EGFP expression in the colon underscores the observations made by flow cytometry (Figure 2). In addition to the *Ahrr*, we analyzed *Cyp1a1* expression in SI tissue after feeding LRD or LRD + I3C. *Cyp1a1* was significantly upregulated in LRD + I3C fed mice (Figure S1e), showing that dietary AhR ligands also upregulate other classical AhR target genes.

Besides dietary constituents, tryptophan metabolites from microbiota have been shown to activate the AhR and drive IL-22 production [5]. In order to assess the influence of the microbiota on AhR activation and *Ahrr* expression in intestinal immune cells, we treated mice for four weeks with broad-spectrum antibiotics to deplete the gut microbiota. Intestinal colony forming units (CFUs) were assessed afterwards (Figure S2e). We observed that antibiotic treatment had no influence on the frequency of AhRR/EGFP^+^ DCs in the mLNs and on AhRR/EGFP^+^ T cells, macrophages, and DCs in the lamina propria of the SI and colon (Figure S2). At most, a small increase in the frequency of AhRR/EGFP^+^ cells in the colonic lamina propria could be observed (Figure S2c). In line, MFI of the AhRR/EGFP reporter was generally not changed by antibiotics treatment across several immune cell subsets in the intestinal immune system. Taken together, our data suggest that AhR activation in intestinal immune cells is mainly driven by dietary factors rather than microbial metabolites.

### 2.3. High Fat Diet and Matching Control Diet Reduce Expression of the AhRR in Intestinal Immune Cells

AIN-93G is a widely used purified diet for rodents and has been used here as a prototypic AhR ligand reduced diet, owing to the absence of phytochemicals and flavonoids with AhR-activating properties [31]. These substances are contained in NC, which is composed of grains, vegetable oil, and mineral/vitamin supplementations. Other commonly used purified diets are, for instance, experimental diets with a high fat content (high fat diet, HFD) to monitor metabolic changes owing to enhanced fat intake.

**Figure 3:**
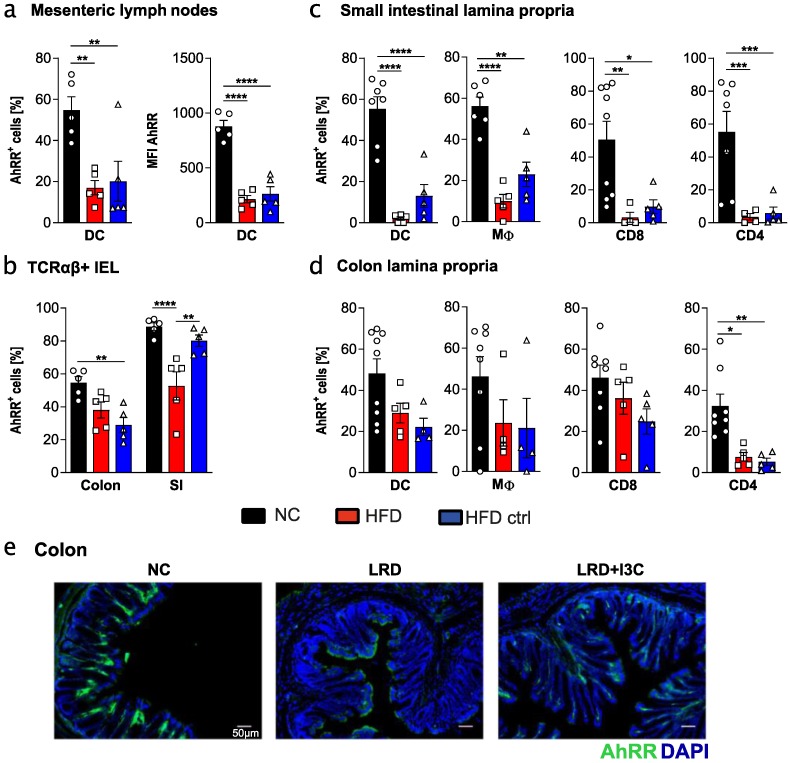
High fat diet and control diet lead to reduced expression of *Ahrr* in intestinal immune cells. AhRR^E/+^ mice were fed for four weeks from weaning onwards with normal chow (NC), high fat diet (HFD), or a matched HFD control diet (HFD ctrl). Frequency of *Ahrr* expressing DCs and MFI of DCs in the mLNs (**a**); frequency of *Ahrr* expressing TCRαβ^+^ IELs (intraepithelial lymphocytes) in colon and small intestine (SI) (**b**); and frequency of *Ahrr* expressing DCs, MΦ, CD4^+^ T cells, and CD8^+^ T cells isolated from the lamina propria of SI (**c**) and colon (**d**). (**e**) After PFA fixation, cryosections of the colon were stained with DAPI in addition to the endogenous EGFP reporter. Data are pooled from at least two independent experiments (*n* = 5–9). Results are shown as mean +/− SEM and significance was analyzed by one-way ANOVA corrected for multiple comparisons by the Tukey’s post hoc test (* *p* < 0.05, ** *p* < 0.01, *** *p* < 0.001, **** *p* < 0.0001). Pictures are representative of at least three different mice from at least two independent experiments.

Here, we investigated whether HFD and the matched control diet (HFD ctrl) [32] affected AhR activation, similar to LRD. We could demonstrate that the frequency of AhRR/EGFP^+^ immune cells in mLN, SI, and colonic lamina propria, as well as in IEL populations, was always lower in HFD- and HFD ctrl-fed mice compared with NC-fed mice, indicating a strongly reduced AhR activating capacity of these diets (Figure 3). This also holds true for the intensity of AhRR/EGFP expression in these cell populations (Figure S3). In most cases, frequencies and MFI of AhRR/EGFP expressing cells were comparable to expression levels after feeding LRD. In small intestinal immune cell populations, frequencies of *Ahrr*-expressing cells were even lower than after LRD feeding (Figure 3c). These data revealed that feeding HFD or HFD ctrl results in significantly lower AhR activation compared with NC. Thus, not only LRD, but also other commonly used purified diets, possess only marginal AhR stimulating activity.

### 2.4. Influence of Dietary AhR Ligands on the Composition of the Intestinal Microbiota

**Figure 4:**
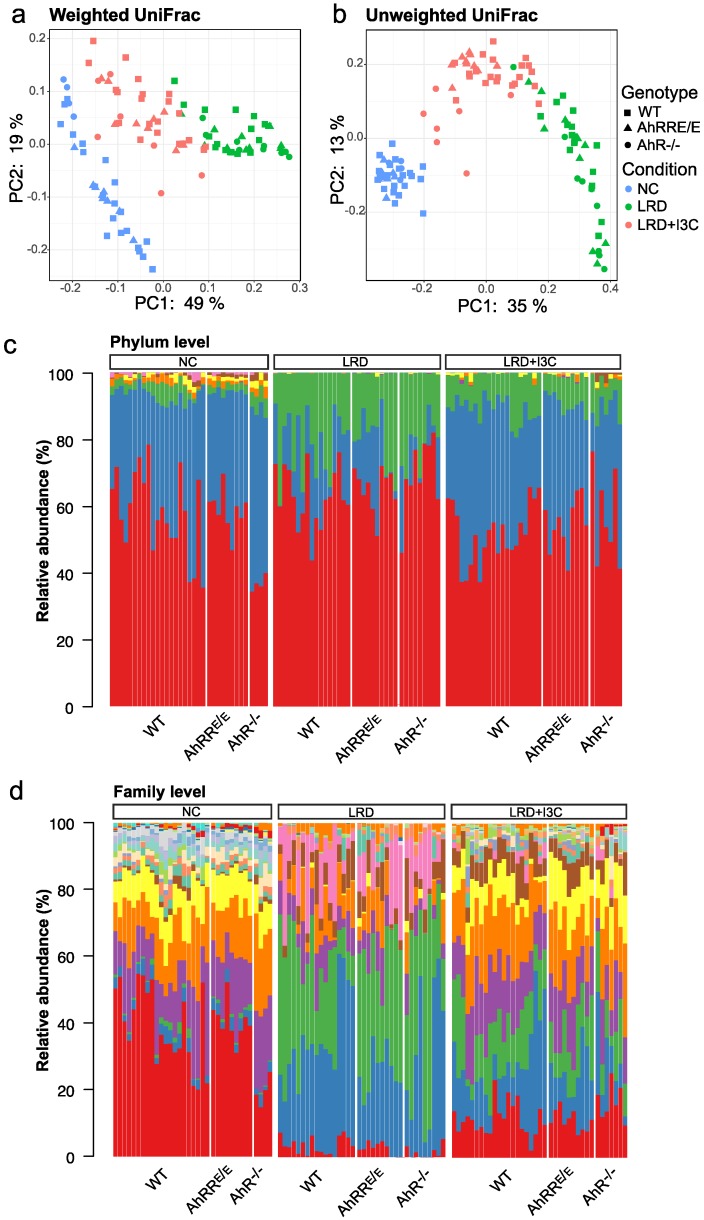
Fecal microbiota differ profoundly between mice fed NC, LRD, or LRD + I3C. Wild-type (WT), AhRR^E/E^, and AhR^−/−^ mice were fed for four weeks from weaning onwards with normal chow (NC), AhR ligand reduced diet (LRD), or LRD supplemented with indole-3-carbinol (LRD + I3C) (2 g/kg). Fecal samples were analyzed by 16S rRNA sequencing. (**a**) Weighted and (**b**) unweighted UniFrac principal component analysis (PCA). (**c**) Relative abundance of microbial communities on phylum level and (**d**) relative abundance of microbial communities on family level. *n* = 21 WT mice fed NC, *n* = 17 WT mice fed LRD, and *n* = 21 WT mice fed LRD + I3C; *n* = 9 AhRR^E/E^ mice fed NC, *n* = 10 AhRR^E/E^ mice fed LRD, and *n* = 10 AhRR^E/E^ mice fed LRD + I3C; *n* = 4 AhR^−/−^ mice fed NC, *n* = 9 AhR^−/−^ mice fed LRD, and *n* = 7 AhR^−/−^ mice fed LRD + I3C.

The data presented above demonstrated that the presence of dietary AhR ligands in NC or LRD + I3C strongly enhanced *Ahrr* expression in intestinal immune cells, whereas depletion of microbiota by antibiotics had no significant influence. Nevertheless, dietary AhR ligands themselves can influence the composition of the microbiome through activation of the AhR pathway in the intestinal mucosa [6,21]. To address this point, we performed a bioinformatic analysis of the microbiome composition after feeding wild-type (WT), AhRR^E/E^, and AhR^−/−^ mice for four weeks with NC, LRD, or LRD + I3C. Fecal samples were analyzed by 16S rRNA sequencing. We first performed UniFrac distance analysis and principal component analysis (PCA) to investigate the general similarities or differences of the microbiome populations. The bacterial composition of the stool samples was clearly different between the dietary conditions (Figure 4a,b).

In contrast, no apparent differences were observed between genotypes in the weighted PCA (Figure 4a), while moderate changes between AhR^−/−^ mice fed LRD + I3C compared with WT and AhRR^E/E^ mice became evident in the unweighted PCA analysis (Figure 4b). In this analysis, all bacterial taxa are counted equally regardless of their abundance in the microbiome, indicating that feeding mice with LRD + I3C induced AhR-dependent changes only in smaller microbial communities of the intestine.

#### 2.4.1. I3C-Induced Changes in the Representation of Bacterial Phyla and Families Are Mostly AhR-Independent

**Figure 5:**
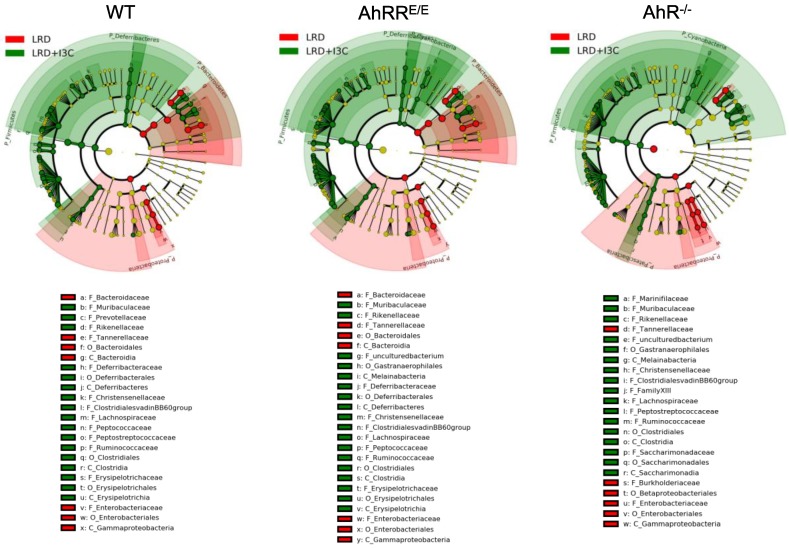
Taxonomic changes in the intestinal microbiome. LEfSe (linear discriminant analysis (LDA) coupled with effect size measurement) was performed to identify taxa displaying the largest differences in abundant microbiota using 16S rRNA sequences. Taxonomix cladograms of WT mice (left), AhRR^E/E^ mice (middle), and AhR^−/−^ mice (right) fed LRD or LRD + I3C. LRD enriched taxa are marked in green and LRD + I3C enriched taxa are marked in red. Taxa with linear discriminant analysis (LDA) scores > 2.0 and *p* < 0.05, determined using Wilcoxon signed-rank test, are shown.

**Figure 6:**
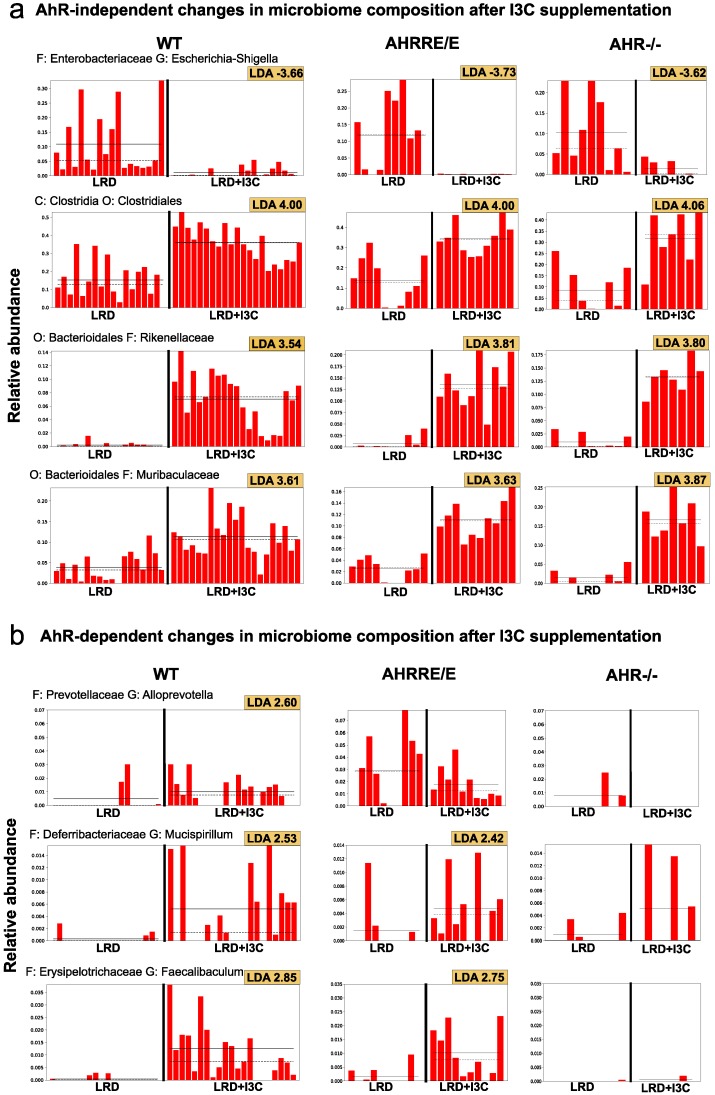
Selection of discriminative biomarkers that are detected by linear discriminant analysis (LDA) coupled with effect size measurements (LEfSe) analysis. Frequencies of biomarkers showing (**a**) AhR-independent changes in microbiome composition after I3C supplementation. Relative abundance of *Escherichia-Shigella* (*Enterobacteriaceae*), *Clostridiales* (*Clostridia*), *Rikenellaceae* (*Bacteriodales*), and *Muribaculaceae* (*Bacteroidales*) are shown. (**b**) AhR-dependent changes in microbiome composition in WT, AhRR^E/E^, and AhR^−/−^ mice fed LRD or LRD + I3C. Relative abundance of *Alloprevotella* (*Prevotellaceae*), *Mucispirillum* (*Deferribacteriaceae*), and *Faecalibaculum* (*Erysipelotrichiaceae*) are presented. Horizontal solid lanes represent the mean, and horizontal dashed lines represent the median of relative abundance of the species. LDA values are depicted in the figures.

We next analyzed the microbial composition in more detail on both phylum and family levels (Figure 4c,d and Figure S4). Changes in microbial populations between LRD and LRD + I3C were also analyzed using linear discriminant analysis (LDA) coupled with effect size measurements (LEfSe) [33] (Figure 5 and Figure 6, Figures S6 and S7). The taxonomic distribution on the phylum level (Figure 4c and Figure S4a) as well as the family level (Figure 4d and Figure S4b) differed markedly between mice fed NC, LRD, and LRD + I3C, with only minor differences between the mouse strains. After feeding LRD, the proportion of *Firmicutes* clearly decreased and *Proteobacteria*, which were only marginally represented in mice fed NC, expanded significantly in all mouse strains analyzed (Figure 4c). On the family level, a clear reduction of *Lachnospiraceae* and *Ruminococcaceae* was visible after feeding LRD. *Bacteroidetes*, *Muribaculaceae*, a dominant family in the mouse intestine previously known as S24-7 [34]; and *Rikenellaceae* dropped in frequency, whereas *Bacteroidaceae* and *Tannerellaceae* increased. The elevated proportion of *Proteobacteriaceae* could be attributed to an expansion of *Enterobacteriaceae* (Figure 4d), which are known to be colitogenic [35]. These data demonstrate that feeding LRD strongly alters the distribution of the bacterial communities in the gut and additionally leads to a major reduction of the Shannon diversity index, indicating a lower alpha diversity (Figure S5).

Addition of I3C to LRD reverted this effect to a great extent (Figure 4, Figure 5 and Figure 6 and Figures S5–S7), which again seemed to be mostly independent of the genotype of the mice. This also becomes apparent by the direct comparison of the microbiome from LRD and LRD + I3C fed mice in the LEfSe analysis (Figure 5). After feeding LRD + I3C, the proportion of *Enterobacteriaceae* was strongly reduced in all three genotypes (LDA scores of about −3.7) and that of *Clostridiales*, here mainly *Lachnospiraceae* and *Ruminococcaceae,* increased (LDA scores of 4.0), which is in line with published data [36] (Figure 6a). Moreover, the frequencies of *Muribaculaceae* and *Rikenellaceae* expanded (LDA scores of 3.5–3.9) (Figure 6a), whereas *Bacteroidaceae* and *Tannerellaceae* were suppressed. In addition, several smaller microbiota communities reappeared, as observed for NC. In line, the alpha diversity of the intestinal microbiome was clearly higher after LRD + I3C, but did not reach the level of NC in WT and AhRR^E/E^ mice. In AhR^−/−^ mice, LRD + I3C brought alpha diversity back to the level of NC, but this level was lower compared with WT and AhRR^E/E^ mice fed NC (Figure S5).

#### 2.4.2. LRD + I3C Also Alters the Frequency of Bacterial Taxa in an AhR-Dependent Manner

When taking a closer look at individual bacterial taxa, certain AhR- and AhRR-dependent changes in the microbiome could also be detected. After feeding LRD, AhRR^E/E^ mice had increased frequencies of *Prevotellaceae (Alloprevotella)* (Figure 6b). In LRD + I3C fed mice, the frequencies of *Erysipelotrichiaceae* were upregulated in WT and AhRR^E/E^ mice, but not in AhR^−/−^ mice (Figure 5 and Figure 6b). Changes in frequencies of *Erysipelotrichiaceae* after I3C supplementation compared with LRD were also previously described by others [37]. Intriguingly, after I3C supplementation, the presence of *Marinifilaceae, Melainabacteria*, and *Saccharimonadaceae* was exclusively upregulated in AhR^−/−^ mice, with LDA scores of 3.13, 2.55, and 2.84, respectively (Figure 5 and Figure S7). In addition, a population of unculturable *Firmicutes* was upregulated in AhR^−/−^ and AhRR^E/E^ mice, but not in WT mice (Figure 5). These significant alterations in microbial communities with an overall minor representation likely account for the differential clustering of the microbiome of AhR^−/−^ mice on I3C diet in the unweighted PCA analysis depicted in Figure 4b.

In summary, we could show that feeding of LRD profoundly changed the microbial composition and reduced the microbial diversity in the intestine. Feeding LRD + I3C reverted these effects to a large extent, but, unexpectedly, the changes in the most abundant bacterial taxa also occurred in AhR-deficient mice and, therefore, were AhR-independent. Besides this, various smaller bacterial communities were significantly regulated in an AhR-dependent manner.

## 3. Discussion

The uptake of dietary AhR ligands exerts important regulatory functions on the integrity of the mucosal barrier, cellular metabolism, and the proper function of the intestinal immune system. Whereas uptake of environmental pollutants such as dioxins may cause liver and immune toxicity [38,39], natural AhR ligands contained in vegetables and fruit represent important constituents of a healthy diet [1,23,40]. Using different AhR ligand-deprived experimental mouse diets and the well-established mouse model of dietary I3C supplementation [6,22], mimicking the consumption of cruciferous vegetables, we here investigated the effect of dietary AhR ligands on AhR activation in various intestinal immune cell subsets. We show that the AhRR/EGFP reporter mouse model is a valuable tool to precisely quantify AhR target gene expression in immune cells in the gut-associated lymphoid tissue (GALT). Interestingly, *Ahrr* expression was predominantly regulated by the presence of dietary AhR ligands, but not by the intestinal microbiota and their metabolites. Although dietary I3C supplementation induced strong expression of the AhRR reporter in myeloid cells and T cells of the lamina propria, as well as IELs and DCs located in the mLNs, many of the profound changes in microbiome composition caused by I3C supplementation also occurred in AhR-deficient mice, and were thus shown to be independent of AhR signalling. Using thorough bioinformatic analyses of these I3C-dependent alterations in the microbiome and direct comparison of WT, AhRR-deficient, and AhR-deficient mice, we were able to identify the microbial communities that responded to I3C stimulation in an AhR-dependent or -independent manner.

Up to now, activation of the AhR through oral ingestion of environmental pollutants or phytochemicals in the intestine has been mainly assessed at the level of AhR target gene expression, with a focus on expression of *Cyp1a1*, the most strongly inducible AhR target gene encoding the cytochrome p450 oxygenase CYP1A1, an important xenobiotic metabolizing enzyme. For this, either RT-qPCR analysis [41] or fluorescent protein reporter mice have been utilized [8]. In the intestine, however, *Cyp1a1* is only expressed in intestinal epithelial cells, but not in intestinal immune cells, and its expression is barely detectable under homeostatic conditions [8]. In contrast, the AhRR/EGFP reporter mouse model offers the possibility to easily quantify the presence of dietary AhR ligands by means of EGFP reporter expression in intestinal immune cells. *Ahrr* expression is readily detectable in mice fed a normal chow diet [21], containing phytochemicals derived from grains and vegetable oils. Remarkably, in mice fed the purified diet AIN-93G (here called LRD), which lacks phytochemicals [31], AhRR expression was reduced by about 5–10-fold based on EGFP reporter expression, indicating a major contribution of food-derived AhR ligands to intestinal AhR activation. Further, we also observed reduced AhRR/EGFP expression in the skin of LRD fed mice, indicating that dietary AhR ligands may also affect distal organ functions (data not shown). As shown in this study, *Ahrr* expression is strongly induced in intestinal immune cells by feeding I3C, which is metabolized to the AhR ligands DIM and ICZ during the gastric passage [42,43]. As demonstrated earlier by other groups and in line with the high susceptibility of AhR^−/−^ mice to development of colitis [4,6,7,21], we also demonstrated an impaired barrier integrity, low IL-22 levels, and increased colitis susceptibility in mice fed LRD. A similar reduction in intestinal *Ahrr* expression as shown for LRD was observed when mice were fed a commercially available HFD or matching control diet. Both HFD and HFD ctrl are purified diets similar to LRD, but differ from LRD regarding carbohydrate composition, and by the addition of a relatively undefined source of fat in the form of lard. Therefore, we would like to point out that commonly used models of diet-induced obesity may be confounded by alterations in intestinal AhR signaling, a problem that is even more pronounced when feeding of HFD is compared with NC rather than HFD ctrl.

In contrast to the dominant role of dietary AhR ligands in regulating *Ahrr* expression in immune cells, we could not detect differences in *Ahrr* reporter expression after depletion of the intestinal microbiota by broad spectrum antibiotics. This does, however, not absolutely exclude a possible participation of microbiota derived AhR ligands in AhR activation in mice fed NC. As the strong reduction in *Ahrr* expression after feeding LRD was accompanied by a major change in microbiota composition, the production of potential AhR ligands by the microbiota may have simultaneously been altered. As an alternative explanation, it is very possible that the composition of the microbiota in our specific-pathogen-free mouse facility does not comprise commensal strains such as *Lactobacillus reuteri*, which have previously been described to be potent producers of AhR activating indoles [5,27,44].

The most unexpected finding of the experiments described in this study was that the I3C induced changes in microbiome composition were to a large extent not AhR-dependent, as they also occurred in AhR-deficient mice and were similar in the AhRR-deficient background. Obviously, I3C stimulation strongly activated the AhR, leading to upregulation of *Ahrr* expression, but this accounted only for a fraction of the alterations in the representation of microbial species. LRD + I3C prevented the outgrowth of potentially colitogenic *Enterobacteriaceae*, which were almost down to the level observed after NC feeding. In addition, the *Clostridiales* families *Lachnospiraceae* and *Ruminococcaceae* were strongly expanded. *Clostridia* are known as major producers of short-chain fatty acids (SCFAs), which are important energy sources for enterocytes and also exert immunoregulatory functions [45]. They additionally support IL-22 production [46], which was markedly increased after I3C supplementation in our colitis experiments. *Clostridia*-produced SCFAs further support the expansion of regulatory T cells, and thus inhibit intestinal inflammation [44,47]. This is a valid explanation for the protection from colitis through I3C, although it appears to be at least partially AhR-independent. In a study very similar to ours, in which the effect of I3C on 2,4,6-trinitrobenzenesulfonic acid-induced colitis was analyzed, an expansion of SCFA-producing *Roseburia spp.* belonging to the *Lachnospiraceae* family was described and correlated with enhanced production of IL-22 and protection form colitis [22]. In this study, however, the dependence of this effect on AhR stimulation was not assessed. Moreover, LRD + I3C did not fully restore the alpha diversity of the microbiota to the complexity seen in NC-fed WT and AhRR^E/E^ mice, which may additionally account for the fact that LRD + I3C fed mice still had a higher susceptibility for DSS colitis than mice fed NC.

In line with our findings, Julliard et al. reported that an I3C-supplemented diet protected both WT and AhR^−/−^ mice from an infection with *Clostridium difficile*, indicating that I3C can act in both an AhR-dependent and -independent manner [48]. It may be important in this context that I3C has been described to possess direct broad-spectrum anti-microbial activity against gram-positive and gram-negative bacteria, including pathogenic *Escherichia coli* at a dose range of 20–80 μg/mL [49]. This concentration could potentially have been reached in the intestine by our feeding regime, although it is unclear to which extent and with which kinetics I3C is metabolized to other compounds during gastric passage. Thus, an anti-microbial activity of I3C may contribute to the loss of *Enterobacteriaceae* observed after feeding LRD + I3C as compared with LRD alone. Another possible mode of action of I3C is via its metabolite DIM, which acts as a ligand not only of the AhR, but also of the orphan G-protein-coupled receptor GPR84 [50]. GPR84 is expressed on immune cells and has been shown to trigger pro-inflammatory signaling pathways and phagocytosis in macrophages [51].

Besides such AhR-independent effects of I3C, our bioinformatic analysis also revealed distinct alterations of the microbiome, which exhibited a clear AhR- or AhRR-dependency. *Alloprevotella*, for example, specifically increased after feeding of LRD + I3C in WT, but not AhR^−/−^ mice. This genus was also detected at a higher frequency in AhRR^E/E^ mice fed LRD compared with WT and AhR^−/−^ mice, possibly indicating a higher AhR activation by residual AhR ligands in LRD-fed mice in the absence of the AhRR. The genus *Mucispirillum* (family *Deferribacteriaceae*) and *Faecalibaculum* (family *Erysipelotrichaceae*), in turn, showed a significant outgrowth in both WT and AhRR^E/E^ mice, but not in AhR^−/−^ mice after feeding LRD + I3C as compared with LRD. Changes in the frequency of *Erysipelotrichaceae* have been associated with AhR signaling in at least two other studies. Brawner et al. showed, however, that *Erysipelotrichaceae* were enriched in the feces of mice fed the LRD AIN-76A, which is slightly different from the LRD used in this study [37]. In addition, feeding of mice with a diet containing broccoli also decreased the abundance of *Erysilelotrichaceae* [23]. In line with our results, on the other hand, a strong reduction of *Erysipelotrichaceae* was associated with a higher susceptibility to inflammatory bowel disease in humans [52,53].

In conclusion, our findings are in agreement with earlier reports that dietary I3C supplementation restores AhR activation in the intestinal mucosa under conditions of malnutrition and deprivation of natural AhR ligands. In humans, such malnutrition may result from a severely reduced consumption of vegetables and fruit in favor of a carbohydrate rich, high fat diet typical of a Western diet, as opposed to a Mediterranean diet. Moreover, in experimental research, it should be taken into consideration that the commonly used HFD products and matching control diets can lead to an impairment of AhR signaling in intestinal immune cells and epithelial cells. On the other hand, we also demonstrate that feeding of LRD with or without I3C supplementation not only affects AhR activation, but may also cause major changes in the composition of the microbiota in an AhR-independent manner, for example, through recognition of dietary constituents by other chemosensing receptors of the host or by direct action on the microbiota itself.

## 4. Materials and Methods

### 4.1. Animals

AhRR^EGFP/EGFP^ (AhRRE/E), heterozygous AhRR^E/+^ mice, and AhR^−/−^ mice [54] were bred at the animal facility of the LIMES, Bonn, Germany. Homozygous AhRR^E/E^ mice are deficient in AhRR expression, and thus represent AhRR-knockout mice. WT littermate mice served as controls. Six- to twelve-week-old male and female mice were used for the experiments and were bred according to German guidelines for animal care. All experiments were performed according to German and Institutional guidelines for animal experimentation (permits: AZ 84-02.04.2011.A186 (approval date: 24 February 2011) and 84-02.04.2016.A210 (approval date: 16 February 2017) and were approved by the government of North Rhine-Westphalia (Germany).

### 4.2. Feeding of Experimental Diets

Mice were fed for four weeks from weaning onwards with different diets. Normal chow diet (NC) was purchased from LASvendi (Soest, Germany), while all other diets were purchased from ssniff Spezialdiäten GmbH (Soest, Germany). AIN 93G1, termed ligand-reduced diet (LRD), was used as purified diet or supplemented with 2 g I3C per kg (LRD + I3C). As an experimental diet with high fat content (HFD), we used ssniff^®^ EF acc. D12492 (I) mod. and ssniff^®^ EF acc. D12450B (I) mod.* was used as control diet, suggested by the vendor. After four weeks of feeding, mice were either analyzed directly or DSS-induced colitis was performed.

### 4.3. Oral Antibiotic Treatment

Mice were treated orally with a broad-sprectrum antibiotic cocktail via the drinking water for four weeks (Ampicillin 1 g/L, Vancomycin 500 mg/L, Ciprofloxacin 200 mg/L, Imipenem 250 mg/L, and Metronidazole 1 g/L in drinking water) in order to deplete the gut microbiota. Depletion was controlled by incubating fecal pellets for 24 h in thioglycolate bouillon, plating them on Columbia blood agar plates and incubating these for another 24 h before analysis. AhRR expression was analyzed in isolated intestinal immune cells by flow cytometry and histologically in colon and mLNs.4.4. DSS-Induced Colitis

Dextran sodium sulfate (DSS) colitis was induced in aged-matched female mice by adding 3% DSS (w/vol) to the drinking water. Mice were kept on DSS-containing water for four days. The general state of health, behavior, posture, stool consistency, rectal bleeding, and body weight were monitored every other day. At the end of the experiment, serum was obtained for analysis of inflammatory cytokines or markers. Small intestine, colon, and mLNs were removed and either transferred into 4% paraformaldehyde (PFA) or stored in phosphate-buffered saline (PBS) on ice until further use. In addition, colon length was determined.

### 4.4. Cytokine Determination in Colon Tissue

After five days of DSS colitis, a small piece of colon tissue was transferred to 500 µL radioimmunoprecipitation assay buffer in a reaction vessel with glass beads and homogenized using the Precellys^®^ 24 tissue homogenizer. The lysate was then transferred to a 1.5 mL Eppendorf tube and centrifuged at 15,000× *g* for 15 min at 4 °C. The supernatant was transferred to a new tube and cytokines were measured by enzyme-linked immunosorbent assay (bio-techne, Wiesbaden, Germany) according to the manufacturer’s instructions.

### 4.5. Determination of Intestinal Permeability

Intestinal barrier function and intestinal barrier permeability were determined after oral application of FITC-coupled Dextran. Mice were fasted 4 h prior to FITC-Dextran administration to facilitate absorption from the intestinal lumen. Mice were orally gavaged with FITC-Dextran in PBS (600 mg per kg body weight). Mice were left without food for a further four hours. Thereafter, serum was sampled and FITC fluorescence was determined with a Tecan infinite M200 plate spectrophotometer at an excitation wavelength of 492 nm and an emission wavelength of 525 nm.

### 4.6. Immunohistology

Tissue samples were fixed for 3 h in 4% PFA at 4 °C and saturated in a sucrose gradient from 5% to 20% sucrose. Samples were embedded in cryomedium and sectioned at a thickness of 10 μm. Sections were counterstained with 0.5 μg/mL DAPI (4,6-diamidino-2-phenylindole). Images were acquired with a Keyence B2900 digital microscope (Keyence Corporation, Osaka, Japan) and analyzed with BZII Analyzer software (Keyence Cooperation).

### 4.7. Isolation of Intestinal Immune Cell Subsets

For isolation of IEL or lamina propria lymphocytes (LPL), the colon and SI were opened longitudinal and cut into 1–2 cm long pieces. To isolate IEL, the tissue was incubated in 15 mM 4-(2-hydroxyethyl)-1-piperazineethanesulfonic acid (HEPES), 5 mM ethylenediaminetetraacetic acid (EDTA), and 10% fetal calf serum (FCS) in PBS for 45 min at 37 °C while shaking. Cells were afterwards filtered through a 70 μM cell strainer. For LPL isolation, mucus removal of intestinal tissue pieces was performed in 5 mM dithiothreitol, 2% FCS, 100 U/mL Penicillin, and 100 ug/mL streptomycin in Hank’s balanced salt solution (HBSS) for 20 min at 37 °C. Epithelial cells were removed by incubation in 5 mM EDTA, 2% FCS, 100 U/mL penicillin, and 100 μg/mL streptomycin in HBSS three times for 15 min at 37 °C and washed with 10 mM HEPES, 100 U/mL penicillin, and 100 μg/mL streptomycin in HBSS for 10 min at 37 °C. Tissue was digested with 4 U/mL liberase and 4000 U/mL DNaseI in 10 mM HEPES, 100 U/mL penicillin, and 100 µg/mL streptomycin in HBSS for 45 min at 37 °C, and afterwards filtered through a 70 μm cell strainer. Cell suspension was centrifuged and stained for analysis by flow cytometry. For preparation of mLN cell suspensions, tissues were meshed through a 100 and 70 μm cell strainer.

### 4.8. Flow Cytometry

Cell suspensions were stained with antibodies against CD3 (145-2C11), CD4 (RM4-5), CD8α (53-6.7), CD8β (YTS156.7.7), MHCII (M5/114.15.2), CD11c (HL-3), F4/80 (CI:A3-1, Abd Serotec, Oxford, UK), CD11b (M1/70), CD64 (X54-5/7.1), TCR β chain (H57-597), TCRγδ (GL3), and Foxp3 (FJK16s). If not indicated otherwise, antibodies were purchased from eBioscience. For intracellular cytokine and transcription factor analysis, cells were fixed with 2% PFA for 20 min, permeabilized with 0.5% saponin in PBS/BSA, and stained for 60 min at room temperature (RT) in the dark. For anti-GFP staining (purified anti-GFP and anti-Rabbit IgG AF488 from Life Technologies), incubation was performed overnight at 4 °C. Cell populations were analyzed with a LSRII Cytometer (BD Biosciences) or a BD FACSymphony™; data were analyzed with FlowJo software (Tree star, Ashland, OR, USA).

### 4.9. Real-Time PCR Analysis

SI tissue was taken from LRD or LRD + I3C fed mice, tissue lysis and homogenization was performed with the Precellys^®^24 homogenizer, and RNA was isolated using the Zymo Research Direct-zol MiniPrep kit according to the manufacturer’s instructions. First-strand cDNA was synthesized from 1 μg of total RNA using Revert Aid reverse transcriptase (Thermo Fisher Scientific, Bonn, Germany). Real-time PCR was performed on a BioRad CFX96 Touch™ Real-Time PCR Detection System using absolute SYBR-green ROX master mix (Thermo Fisher Scientific). Primers were designed using the Universal Probe Library (Roche Applied Science, Mannheim, Germany): cyp1a1 fwd: 5′-CCTCATGTACCTGGTAACCA-3′, cyp1a1 rev: 5′-AAGGATGAATGCCGGAAGGT-3′, GAPDH fwd: 5′-GAGCCAAACGGGTCATCA-3′, GAPDH rev: 5′-CATATTTCTCGTGGTTCACACC-3′.

### 4.10. 16S rRNA Gene Data Collection and Sequencing

Fecal DNA was isolated and purified using the QIAamp DNA Stool Mini Kit (QIAGEN Co., Germany). The V3–V4 hypervariable region of 16S rRNA genes was analyzed according to the Illumina protocol for 16S metagenomic sequencing library preparation with minor modifications. The fecal DNA was amplified with 341F and 806R primers:

Forward:

5′TCGTCGGCAGCGTCAGATGTGTATAAGAGACAGCCTACGGGNGGCWGCAG3′,

Reverse:

5′GTCTCGTGGGCTCGGAGATGTGTATAAGAGACAGGACTACHVGGGTATCTAATCC3′.

PCR amplicons were purified using AMPure XP (Beckman Coulter Co., USA) according to the manufacturer’s instructions. Adapters and barcodes (Nextera XT, Illumina Co., San Diego, CA, USA) were attached to the amplicons to conduct multiplex sequencing. Barcoded amplicons were sequenced using the Illumina MiSeq 2 × 300 bp platform with the MiSeq reagent kit v3 (Illumina Co.) according to the manufacturer’s instructions. The 3′ region of each sequence read with a quality score less than 30 was trimmed using BaseSpace (Illumina Co.).

### 4.11. Microbiome Sequencing Analysis

Raw reads were processed using QIIME2 v.2019.1 with a DADA2 plugin to denoise quality filter reads and call amplicon sequence variants (ASVs), and a feature table of ASV counts was generated. In the quality filtering step, the datasets were truncated to a read length of 270 to 250 base pairs for forward and reverse reads (all other parameters were set to default values). After quality filtering, bacterial taxonomies were assigned to the ASV feature table using the Naïve Bayesian Q2 feature classifier as implemented in QIIME2. Data were compared against a SILVA reference database trained on the V3–V4 region of the 16S rRNA gene. LEfSe analysis was performed to identify taxa displaying the largest differences in abundant microbiota between groups. Only taxa with LDA scores > 2.0 and *p* < 0.05, as determined by Wilcoxon signed-rank test, are shown. All data analyses were performed using R software v3.6.3.

### 4.12. Statistical Analysis

Statistical analysis was performed using the unpaired Student’s *t*-test, if just two independent groups or two conditions on one experimental group were compared. For the determination of any statistical difference between three or more independent experimental groups, one-way analysis of variance (ANOVA) was used. Two-way ANOVA was used when analyzing the mean differences between groups that have been split on two independent variables. All data are presented as mean plus standard error of the mean (SEM) if not stated otherwise in the figure legend. Significance was defined by reaching certain *p*-values in the statistical tests (* *p* < 0.05, ** *p* < 0.01, *** *p* < 0.001, **** *p* < 0.0001). All statistical analyses were conducted with the software GraphPad Prism.

### 4.13. Data Availability

The sequence data for microbiome sequencing were deposited in National Center for Biotechnology Information (NCBI) under the accession number PRJNA615700.

## Supplementary Materials

Supplementary materials can be found at https://www.mdpi.com/1422-0067/21/9/3189/s1.

## Abbreviations

AhR	Aryl hydrocarbon receptor
AhRR	Aryl hydrocarbon receptor repressor
ARNT	AhR nuclear translocator
CFU	Colony forming units
DC	Dendritic cell
DIM	3,3′-Diindolylmethane
DSS	Dextran sodium sulfate
EDTA	Ethylenediaminetetraacetic acid
EGFPFCS	Enhanced Green Fluorescent ProteinFetal calf serum
FITC	Fluorescein isothiocyanate
HBSS	Hank’s balanced salt solution
HEPES	4-(2-hydroxyethyl)-1-piperazineethanesulfonic acid
HFD	High fat diet
HFD ctrl	Matching control diet
I3C	Indole-3-carbinol
IBD	Inflammatory bowel disease
ICZ	indolo[3,2-b]carbazole
IEC	Intestinal epithelial cell
IEL	Intraepithelial lymphocyte
IL	Interleukin
ILC	Innate lymphoid cell
LCN2	Lipocalin-2
LDA	Linear discriminant analysis
LEfSe	LDA coupled with effect size measurements
LPL	Lamina propria lymphocytes
LRD	Ligand-reduced diet
MFI	Mean fluorescent intensity
mLN	Mesenteric lymph node
NC	Normal chow
PAS-B	Period/ARNT/Single-minded
PBS	Phosphate-Buffered Saline
PCA	Principal component analysis
SI	Small intestine
TCR	T cell receptor
WT	Wild-type
