# Supplementary material for: Dietary AhR Ligands Regulate AhRR Expression in Intestinal Immune Cells and Intestinal Microbiota Composition
Source: Int J Mol Sci. 2020 Apr 30;21(9):3189. doi: 10.3390/ijms21093189 (PMC7246938; doi:10.3390/ijms21093189)
Supplement: Supplementary file 1 [file ijms-21-03189-s001.pdf]

Dietary AhR ligands regulate AhRR expression in intestinal immune cells and intestinal microbiota composition

Oliver Schanz [1§], Rieka Chijiiwa [2,3§], Sevgi Can Cengiz [1], Yasmin Majlesain [1], Heike Weighardt [1*], Haruko Takeyama [2,3,4,5*], Irmgard Förster [1*]

1 Immunology and Environment, Life and Medical Sciences (LIMES) Institute, University of Bonn, Carl-Troll-Straße 31, 53115 Bonn, Germany; oliver.schanz@uni-bonn.de (O.S.), secen102@hhu.de (S.C.C.), yasmin_majlesain@uni-bonn.de (Y.M.), heike.weighardt@uni-bonn.de (H.W.), irmgard.foerster@uni-bonn.de (I.F.)

2 Department of Life Science and Medical Bioscience, Graduate School of Advanced Science and Engineering, Waseda University, 2-2 Wakamatsucho, Shinjuku-ku, Tokyo, 162-8480, Japan; rieca.chijiiwa@asagi.waseda.jp (R.C.), haruko-takeyama@waseda.jp (H.T.)

3 Computational Bio Big-Data Open Innovation Laboratory (CBBD-OIL), National Institute of Advanced Industrial Science and Technology, 3-4-1 Okubo, Shinjuku-ku, Tokyo, 169-8555, Japan; rieca.chijiiwa@asagi.waseda.jp (R.C.), haruko-takeyama@waseda.jp (H.T.)

4 Research Organization for Nano and Life Innovation, Waseda University, 513 Wasedatsurumaki-cho, Shinjuku-ku, Tokyo, 162-0041, Japan; haruko-takeyama@waseda.jp (H.T.)

5 Institute for Advanced Research of Biosystem Dynamics, Waseda Research Institute for Science and Engineering, Graduate School of Advanced Science and Engineering, Waseda University, 3-4-1 Okubo, Shinjuku-ku, Tokyo, 169-8555, Japan; haruko-takeyama@waseda.jp (H.T.)

§ equal contribution

* Correspondence: irmgard.foerster@uni-bonn.de; +49 228 73 62780, (I.F.), haruko-takeyama@waseda.jp, Tel.: +81-3-5369-7326, (H.T.), heike.weighardt@uni-bonn.de, +49 228 73 62706, (H.W.)

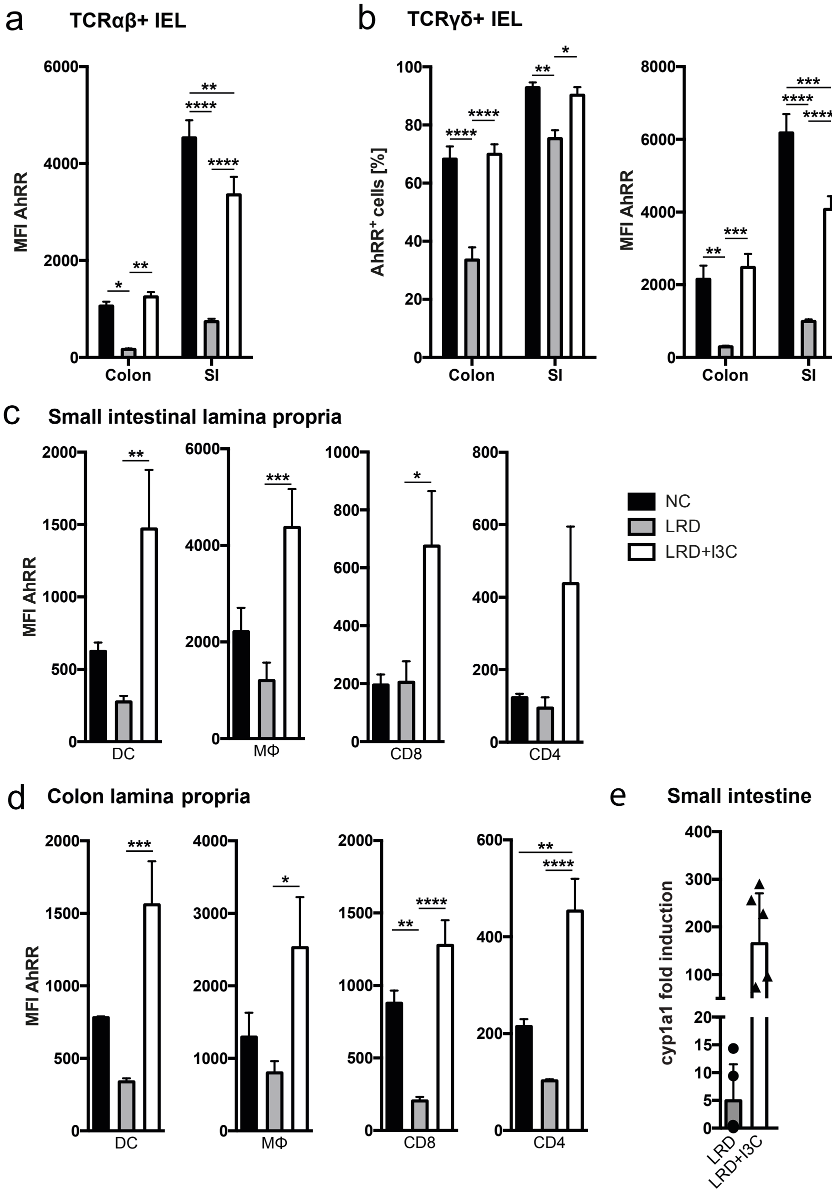

Figure S1: Dietary AhR ligands drive expression of AhRR in intestinal immune cells. AhRR$^{E/+}$ mice were fed for four weeks from weaning onwards with normal chow (NC), AhR ligand reduced diet (LRD) or LRD supplemented with indole-3-carbinol (LRD+I3C) (2 g/kg). (a) MFI of the AhRR in TCR$\alpha\beta^+$ IELs in colon and SI was measured. (b) Frequency of AhRR expressing TCR$\gamma\delta^+$ cells and MFI of the AhRR in TCR$\gamma\delta^+$ cells in colon and SI was determined. MFI of the AhRR in DCs, macrophages, CD8$^+$ T cells and CD4$^+$ T cells from the small intestine lamina propria (c) and colon lamina propria (d) was determined. (e) CYP1A1 expression in SI tissue was determined by qPCR and fold induction was determined by the ddCt method using the housekeeping gene GAPDH. Data is pooled from at least two independent experiments (n=3-5). Results are shown as mean +/– SEM and significance was analyzed by one-way ANOVA corrected for multiple comparisons by the Tukey's Post Hoc Test (*$p<0.05$, **$p<0.01$, ***$p<0.001$, ****$p<0.0001$).

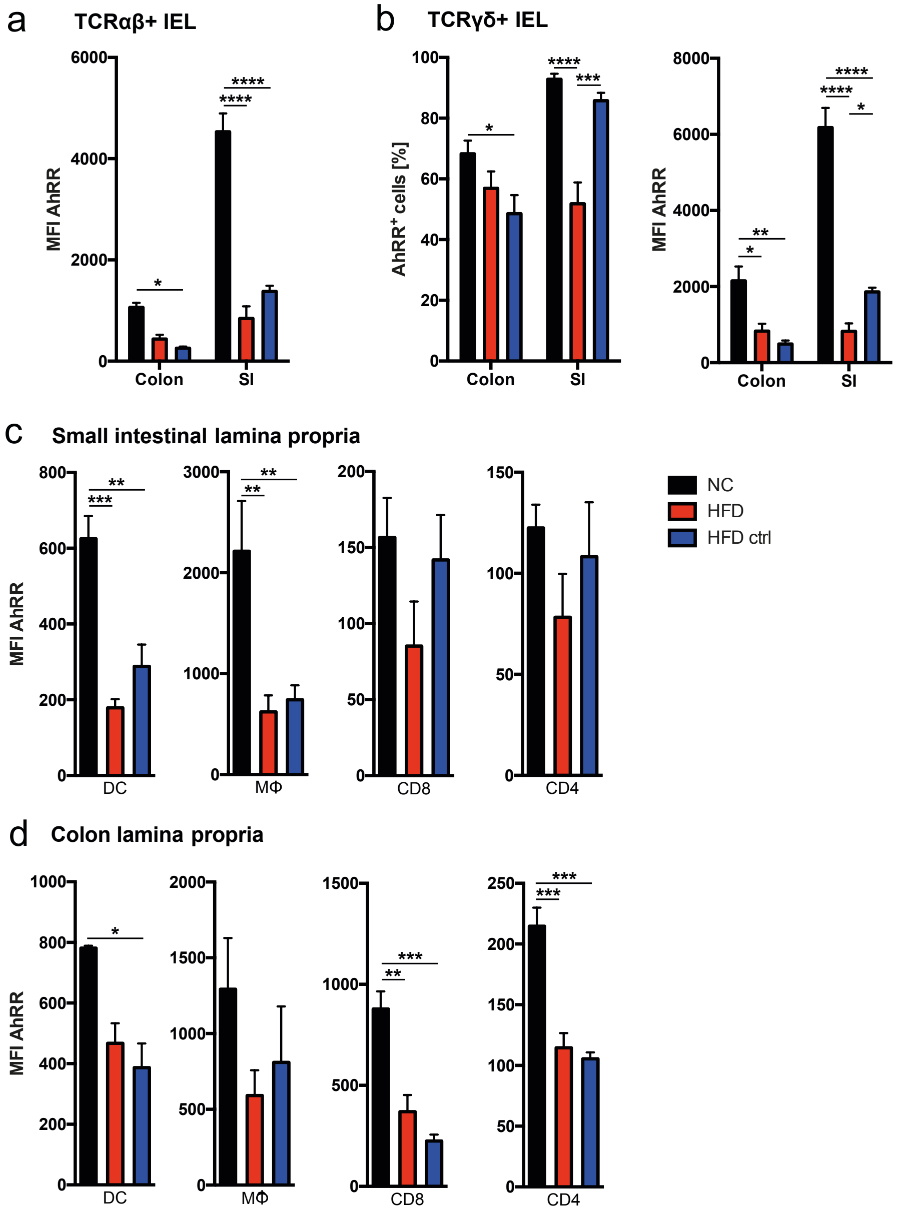

Figure S2: High Fat Diet and control diet lead to reduced expression of AhRR in intestinal immune cells. $AhRR^{E/+}$ mice were fed for four weeks from weaning onwards with normal chow (NC), High Fat Diet (HFD) or a matched HFD control diet. (a) MFI of the AhRR in $TCR\alpha\beta^{+}$ IELs in colon and SI was measured. (b) Frequency of AhRR expressing $TCR\gamma\delta^{+}$ cells and MFI of the AhRR in $TCR\gamma\delta^{+}$ cells in colon and SI was determined. MFI of the AhRR in DCs, macrophages, $CD8^{+}$ T cells and $CD4^{+}$ T cells from the small intestinal lamina propria (c) and colon lamina propria (d) was determined. Data is pooled from at least two independent experiments (n=3-5). Results are shown as mean +/– SEM and significance was analyzed by one-way ANOVA corrected for multiple comparisons by the Tukey's Post Hoc Test (*$p<0.05$, **$p<0.01$, ***$p<0.001$, ****$p<0.0001$).

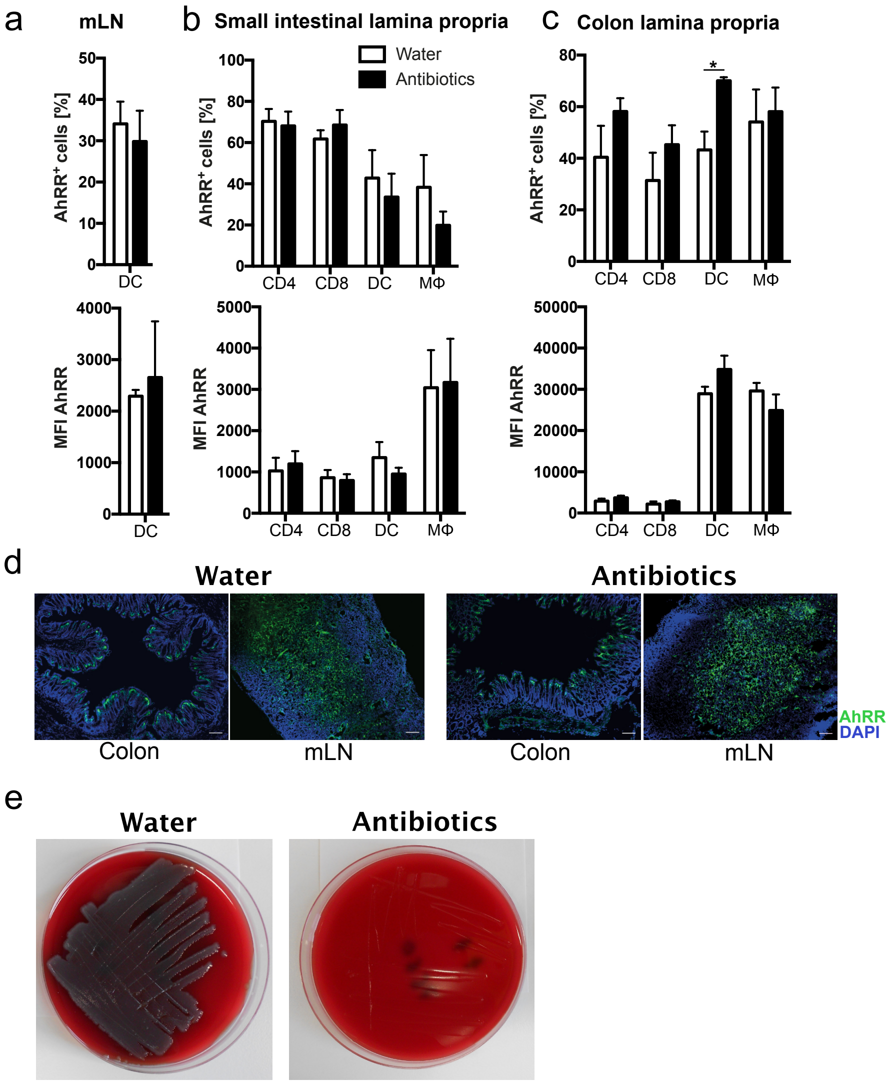

Figure S3: Antibiotic treatment does not influence AhRR expression in intestinal tissue. (a) Frequency of AhRR+ DCs and mean AhRR expression in mLN of AhRR$^{E/+}$ mice after four weeks of antibiotic treatment (Ampicillin 1 g/l, Vancomycin 500 mg/l, Ciprofloxacin 200 mg/l, Imipenem 250 mg/l, Metronidazole 1 g/l in drinking water). CD4+ T cells, CD8+ T cells, DC and MΦ were analyzed for frequency of AhRR+ cells and AhRR MFI in SI (b) and colon (c) of antibiotic-treated and control mice. AhRR expression was analyzed by histology in colon (d) and mLN (e) [scale bar = 100 µm]. Depletion of microbiota was shown by incubating fecal pellets for 24 h in thioglycolate bouillon, plating them on Columbia blood agar plates and incubating these for another 24 h before analysis. One exemplary plate from Ab treated and control mice is shown, respectively. Data is pooled from two independent experiments (n=4 mice) or exemplary shown for one of at least two independent experiments (MFI). Results are shown as mean +/– SEM and significance was determined by two-tailed student's t-test corrected for multiple comparisons by the Holm-Sidak method (*p<0.05).

a

- D_0__BacterialD_1__Bacteroidetes
- D_0__BacterialD_1__Firmicutes
- D_0__BacterialD_1__Proteobacteria
- D_0__BacterialD_1__Actinobacteria
- D_0__BacterialD_1__Cyanobacteria
- D_0__BacterialD_1__Deferribacteres
- D_0__BacterialD_1__Patescibacteria
- D_0__BacterialD_1__Tenericutes
- D_0__BacterialD_1__Verrucomicrobia
- D_0__Bacterial__
- Unassignedl__

b

- D_0__BacterialD_1__BacteroidetesID_2__BacteroidialD_3__BacteroidalesID_4__Muribaculaceae
- D_0__BacterialD_1__BacteroidetesID_2__BacteroidialD_3__BacteroidalesID_4__Bacteroidaceae
- D_0__BacterialD_1__BacteroidetesID_2__BacteroidialD_3__BacteroidalesID_4__Tannerellaceae
- D_0__BacterialD_1__FirmicutesID_2__ClostridialD_3__ClostridialesID_4__Lachnospiraceae
- D_0__BacterialD_1__FirmicutesID_2__ClostridialD_3__ClostridialesID_4__Ruminococcaceae
- D_0__BacterialD_1__BacteroidetesID_2__BacteroidialD_3__BacteroidalesID_4__Rikenellaceae
- D_0__BacterialD_1__ProteobacterialD_2__DeltaproteobacterialD_3__DesulfovibrionalesID_4__Desulfovibrionaceae
- D_0__BacterialD_1__ProteobacterialD_2__GammaproteobacterialD_3__EnterobacterialesID_4__Enterobacteriaceae
- D_0__BacterialD_1__BacteroidetesID_2__BacteroidialD_3__BacteroidalesID_4__Prevotellaceae
- D_0__BacterialD_1__ProteobacterialD_2__GammaproteobacterialD_3__BetaproteobacterialesID_4__Burkholderiaceae
- D_0__BacterialD_1__FirmicutesID_2__ClostridialD_3__ClostridialesID_4__Peptostreptococcaceae
- D_0__BacterialD_1__FirmicutesID_2__ClostridialD_3__ClostridialesID_4__Clostridiales vadinBB60 group
- D_0__BacterialD_1__FirmicutesID_2__ErysipelotrichialD_3__ErysipelotrichalesID_4__Erysipelotrichaceae
- D_0__BacterialD_1__ActinobacterialD_2__ActinobacterialD_3__BifidobacterialesID_4__Bifidobacteriaceae
- D_0__BacterialD_1__ActinobacterialD_2__CoriobacteriialD_3__CoriobacterialesID_4__Atopobiaceae
- D_0__BacterialD_1__ActinobacterialD_2__CoriobacteriialD_3__CoriobacterialesID_4__Eggerthellaceae
- D_0__BacterialD_1__BacteroidetesID_2__BacteroidialD_3__BacteroidalesID_4__Marinifilaceae
- D_0__BacterialD_1__BacteroidetesID_2__BacteroidialD_3__Bacteroidalesl__
- D_0__BacterialD_1__CyanobacterialD_2__MelainabacterialD_3__GastranaerophilalesID_4__uncultured bacterium
- D_0__BacterialD_1__CyanobacterialD_2__OxyphotobacterialD_3__ChloroplastID_4__Pyropia yezoensis
- D_0__BacterialD_1__CyanobacterialD_2__OxyphotobacterialD_3__Chloroplastl__
- D_0__BacterialD_1__DeferribacteresID_2__DeferribacteresID_3__DeferribacteralesID_4__Deferribacteraceae
- D_0__BacterialD_1__FirmicutesID_2__BacillilD_3__BacillalesID_4__Planococcaceae
- D_0__BacterialD_1__FirmicutesID_2__BacillilD_3__LactobacillalesID_4__Enterococcaceae
- D_0__BacterialD_1__FirmicutesID_2__BacillilD_3__LactobacillalesID_4__Lactobacillaceae
- D_0__BacterialD_1__FirmicutesID_2__BacillilD_3__LactobacillalesID_4__Streptococcaceae
- D_0__BacterialD_1__FirmicutesID_2__ClostridialD_3__ClostridialesID_4__Christensenellaceae
- D_0__BacterialD_1__FirmicutesID_2__ClostridialD_3__ClostridialesID_4__Clostridiaceae 1
- D_0__BacterialD_1__FirmicutesID_2__ClostridialD_3__ClostridialesID_4__Defluviitaleaceae
- D_0__BacterialD_1__FirmicutesID_2__ClostridialD_3__ClostridialesID_4__Family XIII
- D_0__BacterialD_1__FirmicutesID_2__ClostridialD_3__ClostridialesID_4__Peptococcaceae
- D_0__BacterialD_1__FirmicutesID_2__ClostridialD_3__Clostridialesl__
- D_0__BacterialD_1__Firmicutesl__l__l__
- D_0__BacterialD_1__PatescibacterialD_2__SaccharimonadialD_3__SaccharimonadalesID_4__Saccharimonadaceae
- D_0__BacterialD_1__ProteobacterialD_2__AlphaproteobacterialD_3__RhizobialesID_4__Rhizobiaceae
- D_0__BacterialD_1__ProteobacterialD_2__AlphaproteobacterialD_3__RhodospirillalesID_4__uncultured
- D_0__BacterialD_1__ProteobacterialD_2__AlphaproteobacterialD_3__RickettsialesID_4__Mitochondria
- D_0__BacterialD_1__ProteobacterialD_2__Deltaproteobacterial__l__
- D_0__BacterialD_1__ProteobacterialD_2__GammaproteobacterialD_3__VibrionalesID_4__Vibrionaceae
- D_0__BacterialD_1__ProteobacterialD_2__GammaproteobacterialD_3__XanthomonadalesID_4__Xanthomonadaceae
- D_0__BacterialD_1__Proteobacterial__l__l__
- D_0__BacterialD_1__TenericutesID_2__MollicutesID_3__AnaeroplasmatalesID_4__Anaeroplasmataceae
- D_0__BacterialD_1__TenericutesID_2__MollicutesID_3__Mollicutes RF39ID_4__metagenome
- D_0__BacterialD_1__TenericutesID_2__MollicutesID_3__Mollicutes RF39ID_4__uncultured Lachnospiraceae bacterium
- D_0__BacterialD_1__TenericutesID_2__MollicutesID_3__Mollicutes RF39ID_4__uncultured bacterium
- D_0__BacterialD_1__TenericutesID_2__MollicutesID_3__Mollicutes RF39l__
- D_0__BacterialD_1__VerrucomicrobialD_2__VerrucomicrobiaeID_3__VerrucomicrobialesID_4__Akkermansiaceae
- D_0__Bacterial__l__l__l__
- Unassignedl__l__l__l__

Figure S4: Fecal microbiota differs profoundly from NC after feeding LRD or LRD+I3C. Fecal microbial composition of mice fed with normal chow (NC), AhR ligand reduced diet (LRD) or LRD supplemented with indole-3-carbinol (LRD+I3C) (2 g/kg). (a) Legend for Figure 4a: relative abundance of fecal microbial communities on a phylum level. (b) Legend for Figure 4b: relative abundance of fecal microbial communities on family level.

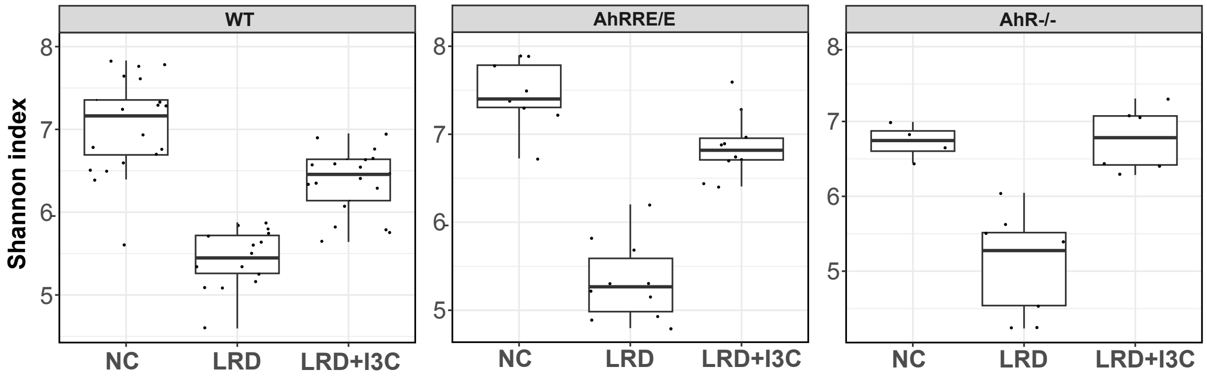

Figure S5: Distribution of alpha diversity as measured by Shannon index. Box plots represent the Shannon index for fecal microbial communities of WT mice (right), AhRR$^{E/E}$ mice (middle), AhR$^{-/-}$ mice (left) fed normal chow (NC), AhR ligand reduced diet (LRD) or LRD supplemented with indole-3-carbinol (LRD+I3C).

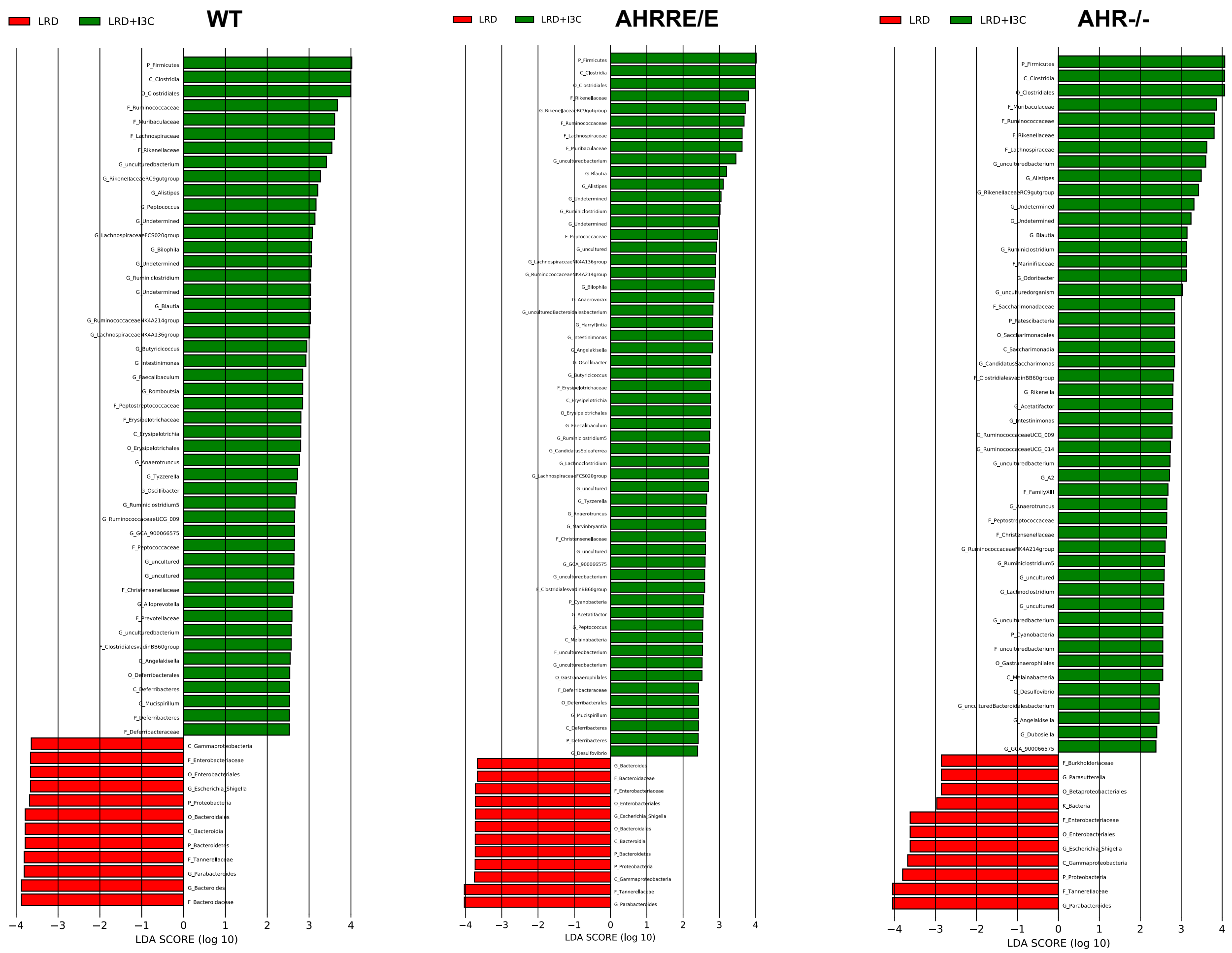

Figure S6: Taxonomic changes in the intestinal microbiome. Linear discriminant analysis (LDA) scores for abundant taxa between AhR ligand reduced diet (LRD) or LRD supplemented with indole-3-carbinol (LRD+I3C) in WT mice (right), AhRR$^{E/E}$ mice (middle), AhR$^{-/-}$ mice (left). Discriminative biomarkers with LDA scores > 2.0 and $p < 0.05$, determined using Wilcoxon signed-rank test, are shown.

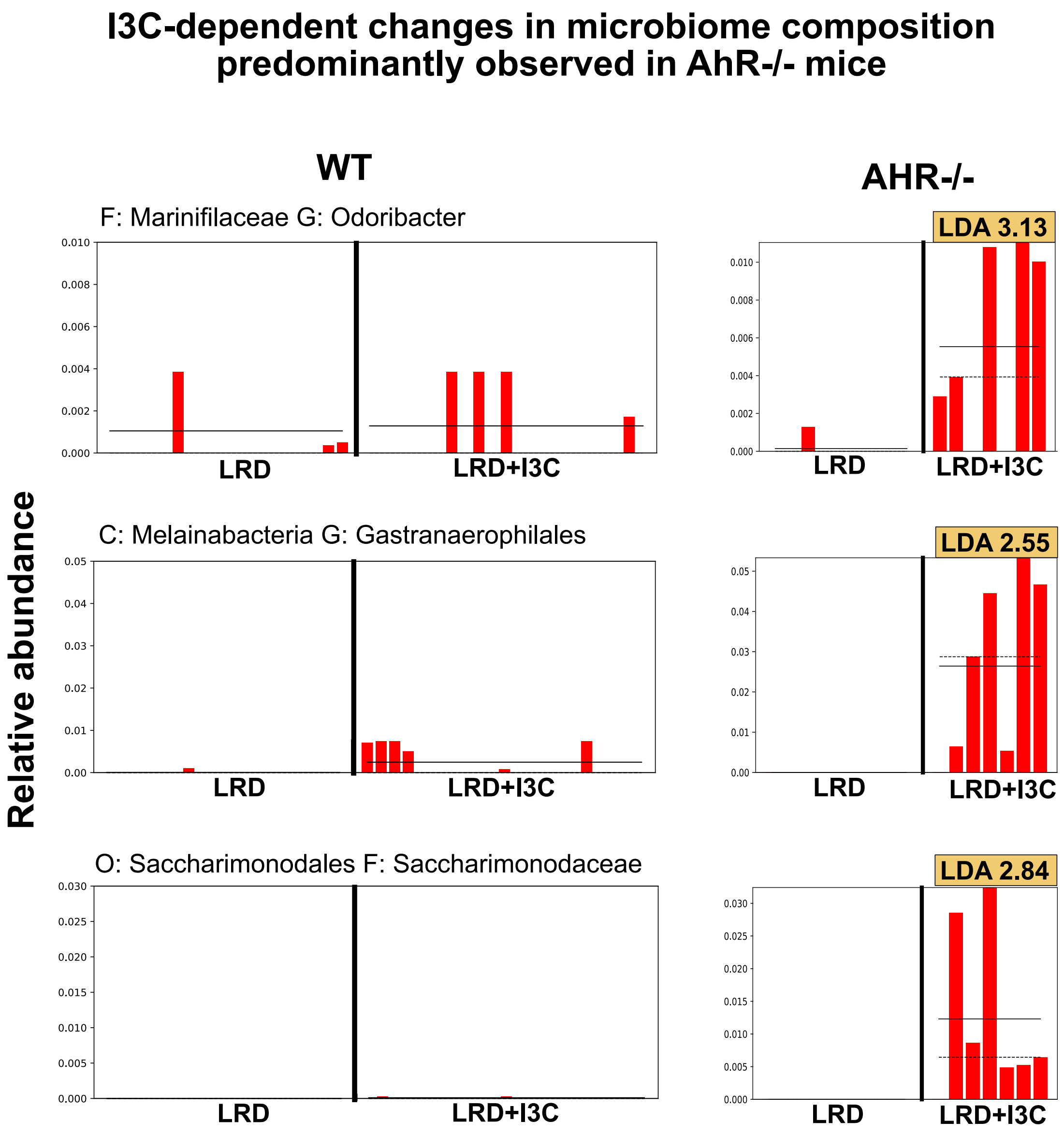

Figure S7: Selection of discriminative biomarkers based on LEfSe analysis. Frequencies of biomarkers showing I3C-dependent changes in microbiome composition predominantly observed in $AhR^{-/-}$ mice after feeding WT, $AhRR^{E/E}$ and $AhR^{-/-}$ mice AhR ligand reduced diet (LRD) or LRD supplemented with indole-3-carbinol (LRD+I3C). Relative abundance of *Odoribacter* (*Marinifiliaceae*), *Gastranaerophilales* (*Melainabateria*) and *Saccharimonodaceae* (*Saccharimonodales*) are depicted. Horizontal solid lanes represent the mean, horizontal dashed lines represent the median of relative abundance of the species. Linear Discriminant Analysis (LDA) values are depicted in the figures.
